# Precise Surface State Control of Carbon Quantum Dots to Enhance Charge Extraction for Solar Cells

**DOI:** 10.3390/nano10030460

**Published:** 2020-03-04

**Authors:** Qiming Yang, Wen Yang, Yong Zhang, Wen Ge, Xin Yang, Peizhi Yang

**Affiliations:** 1 Key Laboratory of Advanced Technique & Preparation for Renewable Energy Materials, Ministry of Education, Yunnan Normal University, Kunming 650092, China; yqm920919@163.com (Q.Y.); gewen1024@126.com (W.G.); yangxinzju@zju.edu.cn (X.Y.); 2 Department of Electrical and Computer Engineering and Center for Optoelectronics, University of North Carolina at Charlotte, 9201 University City Blvd, Charlotte, NC 28223, USA; yong.zhang@uncc.edu

**Keywords:** nitrogen-doped carbon quantum dots, co-sensitized solar cell, up-convention, light absorption

## Abstract

Dye-sensitized solar cells are regarded as promising candidates to resolve the energy and environmental issues in recent years, arising from their solution-processable fabrication technology and high power conversion efficiency. However, there are still several problems regarding how to accelerate the development of this type of photovoltaics, including the limited light-harvesting ability and high-production cost of molecular dye. In the current work, we have systematically studied the role of nitrogen-doped carbon quantum dots (N-CQDs) as co-sensitizers in traditional dye sensitized solar cells. A series of N-CQDs have been prepared by employing chitosan as a precursor via one-pot hydrothermal technology for various times, demonstrating a maximized efficiency as high as 0.089% for an only N-CQDs-based device. Moreover, the co-sensitized solar cell based on N719 dye (C_58_H_86_N_8_O_8_RuS_2_) and optimized N-CQDs shows significantly enhanced performance, yielding a solar-to-electric conversion efficiency of up to 9.15% under one standard sun (AM 1.5G) irradiation, which is much higher than the 8.5%-efficiency of the controlled device without N-CQDs. The matched characteristics of energy level, excellent up-convention, and FRET (Förster resonance energy transfer) abilities of N-CQDs are responsible for their improved power conversion efficiency.

## 1. Introduction

In the past decades, dye-sensitized solar cells (DSSCs) have been considered as a novel alternative to silicon-based photovoltaics due to their low cost and relatively high conversion efficiency [1]. However, the expensive transition metals element (such as Ru complexes) and limited light-absorption range of traditional dye molecules [2] are believed to be a great challenge for the further development of DSSCs in the future. To solve these issues, some inorganic quantum dots (QDs) have been used as a replacement for dye molecules or combined with dye molecules as photosensitizers. Importantly, the co-sensitization can compensate the narrow absorption spectrum of the dye molecule as well as realize the synergistic interaction between the QDs and dye molecules, significantly enhancing the light-absorption capability of the overall photovoltaic device [3]. However, the metal chalcogenide QDs, such as Cd-containing and Pb-containing quantum dots, are typically used in quantum dots sensitized solar cells (QDSCs), which limits the practical applications of the corresponding solar cells because of health and environmental issues [4]. Hence, environmentally friendly alternative QDs are extremely desirable and welcomed to fabricate the state-of-the-art QDSCs.

The emerging carbon quantum dots (CQDs) as a kind of fascinating carbon nanomaterials have attracted tremendous interest due to their nature semiconducting properties, especially the good photo-induced electron transfer ability and large two-photon absorption cross-section [5]. Recently, N doping has been demonstrated as a promising strategy to tune the optical properties and energy band structure of CQDs for application in opto-electronic devices, presenting improved solar-to-electric conversion efficiency upon combining with a TiO_2_ electrode [6]. As a result, they are expected to display enhanced performance for solar cells as well. In recent years, the synthesis of fluorescent CQDs generally adopts some economic and environmental-friendly materials and methods, such as watermelon peel [7], orange juice [8], strawberry powder [9], glucose [10], chitosan [11,12], the hydrothermal method [8,9,10], the microwave method [13], and the ultrasonic method [14]. Note that chitosan is a derivative of chitin (a natural rich glycosaminoglycan), containing a large amount of functional groups such as aminos (–NH_2_) and hydroxyls (–OH) in their chemical structure. Chitosan is a kind of natural non-toxic material, and it shows great potential to be an excellent precursor to obtain high-quality N-CQDs [13].

Therefore, in this work, we employed chitosan powders as a precursor to synthesize water-soluble photoluminescent CQDs via hydrothermal treatment. According to the elemental analysis, we found that the CQDs can be successfully doped by N atoms under high temperature and pressure, which are denoted as the N-CQDs. Initially, the synthesized N-CQDs are used as photo-sensitizers to assemble quantum dot sensitized solar cells (QDSCs), exhibiting excellent optical properties. However, the fabricated QDSCs with N-CQDs alone yielded very lower power conversion efficiencies (PCEs) of <0.09% owing to the weak affinity between CQDs and photoanode TiO_2_ [14,15]. Aiming to obtain higher efficiency, we fabricated a co-sensitized solar cell with dye-N719 (C_58_H_86_N_8_O_8_RuS_2_, the chemical structure is provided in Figure S1) and optimized N-CQDs, yielding a champion PCE of 9.15%, which is significantly higher than 8.5% for the device without N-CQDs.

## 2. Results and Discussion

**Figure 1:**
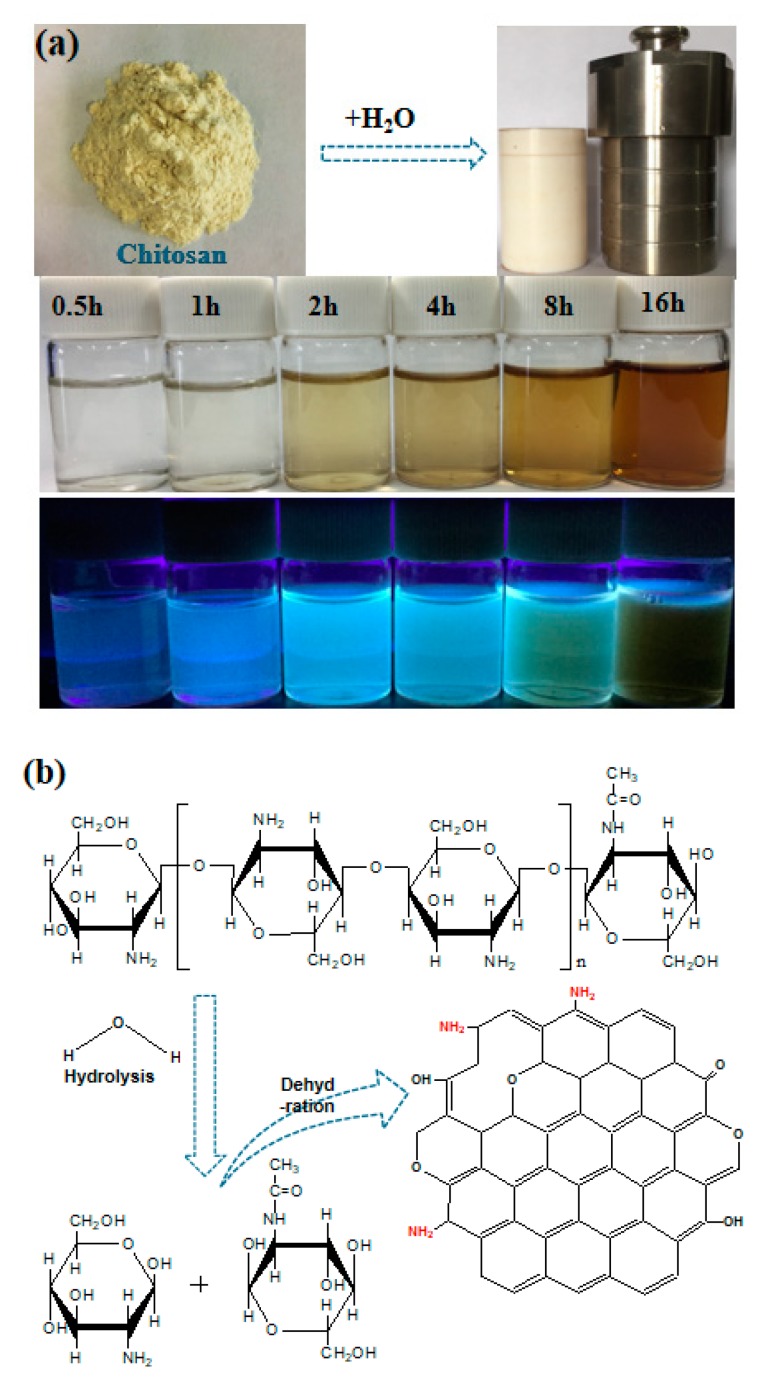
(**a**) Conversion processes from chitosan powders to carbon quantum dots (CQDs) by a hydrothermal method and images of nitrogen-doped carbon quantum dots (N-CQDs) aqueous solutions synthesized at different heating times under UV light irradiation. (**b**) Synthesis strategies of N-CQDs from chitosan.

As illustrated in Figure 1a, using chitosan powders as precursors, N-CQDs was synthesized via a one-step hydrothermal method by varying the heating time ranging from 0.5 to 16 h, and the reaction temperature is controlled at 180 °C. It is interesting that the coloration of the resultant N-CQDs solution is clearly observed in Figure 1a, suggesting an increased N-CQD concentration along with increasing the treatment time, which is mainly attributed to the formation of more nucleus and the growth of previously formed CQDs. Therefore, it can be predicted that the size of as-prepared CQDs can be easily tuned by simply changing the synthesis time and therefore the corresponding solar-to-electric properties, which can be cross-checked by the photoluminescence behaviors (see Figure 1a). Upon excitation by a 365 nm ultraviolet (UV) lamp, all N-CQD solutions display bright cyan photoluminescence with different intensities. The mechanism behind this conversion (Figure 1b) is that the major ingredient of chitosan is glycosaminoglycan, which will be hydrolyzed into glucosamine during the hydrothermal reaction process. The intermediate glucosamine is further converted into N-CQDs by the subsequent dehydration and *N*-condensation reaction [16] showing the feasibility to obtain N-CQDs via this one-step hydrothermal method. During this process, the nitrogen element can be introduced into the C=C functional group under high temperature owing to the presence of the amino group, which is benefit for the modulation of the electron distribution and therefore the energy level architecture. Until now, it can be concluded that the reaction time plays a key role in determining the final properties of the as-prepared N-CQDs.

**Figure 2:**
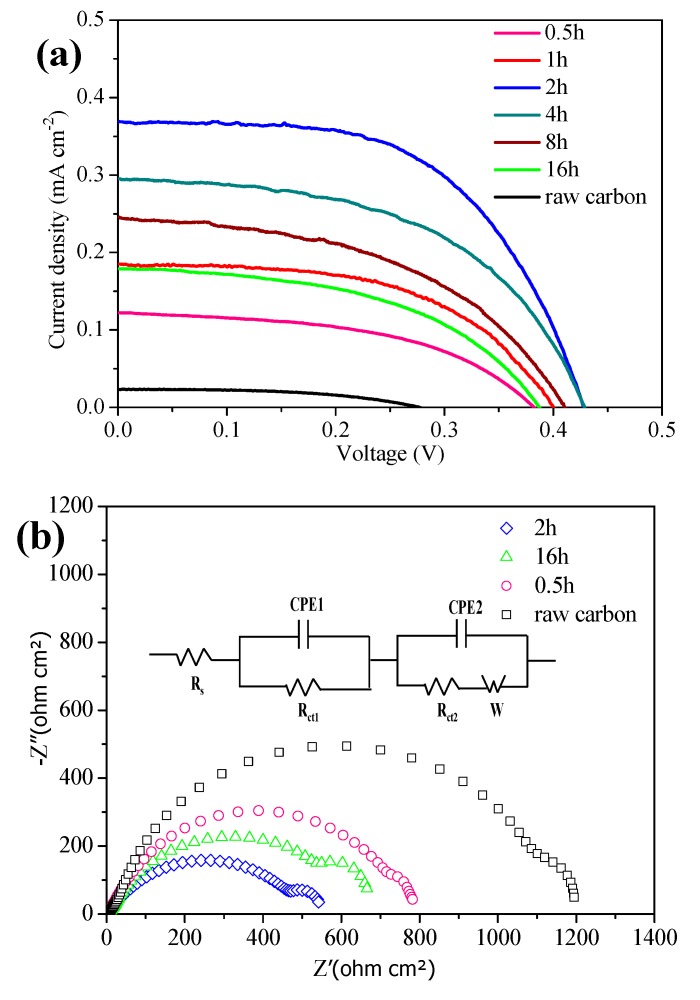
(**a**) Characteristic photocurrent density–voltage (*J*–*V*) curves for N-CQDs sensitized solar cells under simulated sunlight (AM1.5, 100 mW cm^−2^), (**b**) Nyquist electrochemical impedance spectroscopy (EIS) plots and an equivalent circuit for N-CQDs sensitized solar cells.

To better understand the effect of reaction time on the final photovoltaic performance of CQDs, a serious of N-CQDs sensitized TiO_2_-based solar cells are assembled and characterized. The corresponding photocurrent density voltage (*J*–*V*) curves are recorded as shown in Figure 2a, and the photovoltaic parameters including short-circuit current density (*J*_sc_), open-circuit voltage (Voc), fill factor (FF), and PCE are summarized in Table 1. To prove the performance enhancement of the device based on N-CQDs, the efficiency curve obtained from the raw activated carbon is provided in Figure 2a. Obviously, the device tailored by N-CQDs at 2 h heating time yields a maximal PCE of 0.089% with a *J*_sc_ of 0.369 mA cm^−2^, *V*_oc_ of 0.508 V, and FF of 56% under AM 1.5 (100 mW cm^−2^) irradiation. The results suggest that the N-CQDs at 2 h heating time have better ability for light absorption and electron transfer rate. The underlying mechanism can be attributed to the incomplete honeycomb graphene structure and weak electron transport ability for N-CQDs fabricated at a shorter heating time, while the quantum effects of N-CQDs will be weakened upon prolonging the time [9]. The high-resolution TEM micrograph of N-CQDs at 0.5 h, 2 h, and 8 h heating times are given in Figure S2, these images examine the rationality of the above explanation. Table S1 provide detailed data about as-prepared CQDs in comparison to other kinds of carbon dots sensitized solar cells reported previously as light absorbers, and the efficiency achieved in this paper is moderate compared to the efficiencies in the literature. Meanwhile, 15 devices based on various electrolytes have been measured to demonstrate the repeatability, and the error bars of the efficiency calculations are given in Figure S3. Taking the fabrication technology and efficiency into consideration simultaneously, the self-doping N-CQDs via the one-step hydrothermal method are promising to accelerate the further development of QDSCs.

Furthermore, the electrochemical impedance spectroscopy (EIS) measurements are used to explore the interfacial charge transfer dynamics of the device, as shown in Figure 2b. The corresponding electrochemical parameters including the series resistance (Rs), interfacial charge transfer resistance at the Pt_3_Ni/electrolyte interface (Rct1), and the N-CQDs/TiO_2_/electrolyte interface (Rct2) are fitted by an equivalent circuit (which has been inserted into Figure 2b) and summarized in Table S2. Obviously, Rct2 has a minimum value (200 ohm cm^2^) for the device sensitized with N-CQDs at 2 h heating time, an indicator of smaller charge transfer resistance between the N-CQDs/TiO_2_ and an electrolyte interface, which is mainly attributed to the interaction between the surface-grouped N atom and O atom on the surface of TiO_2_. In this manner, an enhanced carrier concentration in the conduction band of TiO_2_ and the increased potential difference can be realized, which is beneficial for promoting a photo-generated carrier transfer to the counter electrode by percolating through mesoscopic TiO_2_ pathways [17] and leading to a larger *J*_sc_ as well as better power efficiency.

**Figure 3:**
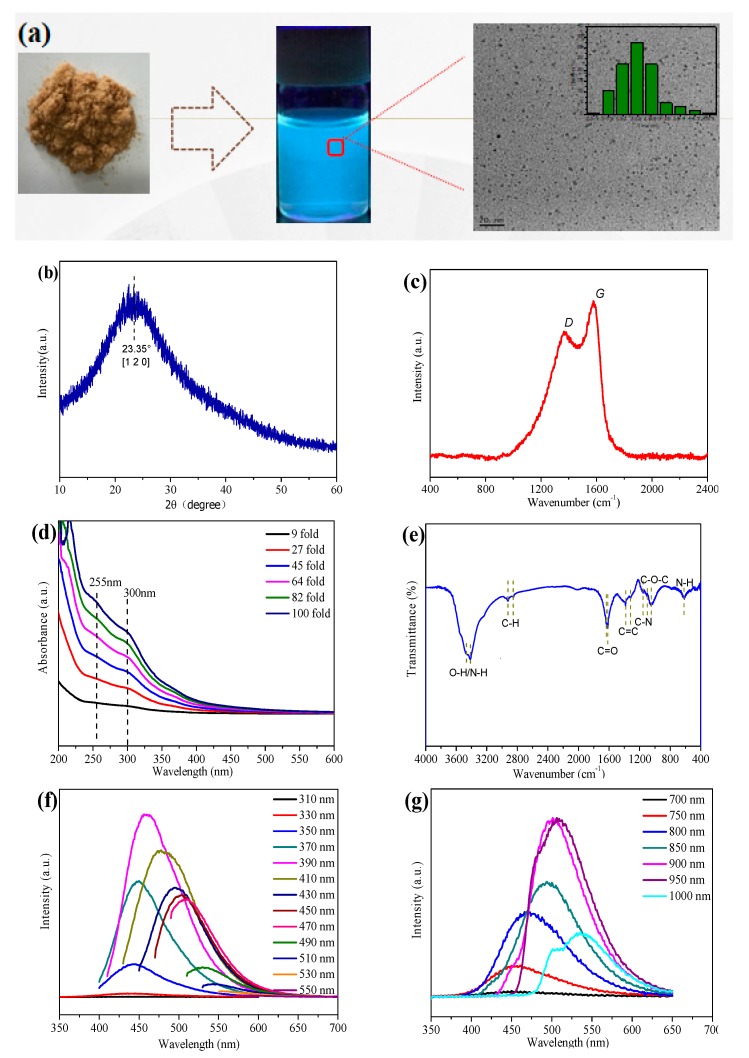
(**a**) Quantitative dispersion from solid-state N-CQDs to N-CQDs solution; (**b**) X-ray diffraction pattern of N-CQDs; (**c**) Raman spectrum of N-CQDs; (**d**) UV-vis absorption spectra of N-CQDs; (**e**) Fourier Transform Infrared (FTIR) spectrum of N-CQDs; (**f**) photoluminescence (PL) spectra of the N-CQDs; (**g**) Up-convention fluorescence spectra of N-CQDs; The heating time for N-CQDs is 2 h.

According to the above-mentioned discussion, it can be obviously seen that the reaction time plays a key role in determining the final photovoltaic conversion capacity of N-CQDs. To highlight the intrinsic optical behavior of N-CQDs, the sample at 2 h heating time is purified via freeze-dried and then dispersed in ethanol solution with a concentration of 50 mg mL^−1^, as shown in Figure 3a. The optimized N-CQDs exhibit brighter cyan photoluminescence under 365 nm UV light, suggesting an effective photo-induced charge transfer mechanism and reduced defect-trapped charger no-irradiation recombination, in other words, optimized quantum yield. When the heating time is shorter or prolonged, the half-baked conjugated structure or oversized quantum dots can be formed, respectively, leading to inferior photoluminescence. The low-resolution TEM graph of the corresponding N-CQDs is shown in Figure 3a, demonstrating that the N-CQDs are well-dispersed spherical dots. The size distribution is plotted as the inset of Figure 3a, with a size distribution ranging from 1.5 to 3 nm and an average size of 2 nm. A high-resolution TEM image of N-CQDs is given in Figure S4 with interplanar spacing of 0.24 nm. All the characteristics together demonstrate the successful formation of high-quality carbon quantum dots.

To better understand the structure of N-CQDs, the X-ray diffraction (XRD) pattern is characterized as shown in Figure 3b. There is a broad characteristic peak at 2θ = 22.35°, which is assigned to the peak of graphene, indicating an amorphous carbon phase for N-CQDs [18]. Additionally, the Raman spectrum (Figure 3c) shows two characteristic peaks around 1370 (D band) and 1503 cm^−1^ (G band), where the D band is connected with defects of the carbon based areas, whereas the latter is related to the vibration of sp^2−^binding carbon atoms in a 2D hexagonal lattice [19]. The relative intensity ratio of D and G has been calculated to be about 0.81, revealing the presence of defects, edges and vacancies in N-CQDs [20].

Figure 3d shows the UV-Vis absorption spectra of the various concentrated N-CQDs aqueous solutions. There is a fact that the peak absorption intensity is highly dependent on the solution concentration and the peak position is nearly unchanged, demonstrating a linear relationship with respect to the increased solution concentration. The absorption spectrum of N-CQDs shows two characteristic absorption peaks located at 255 nm and 300 nm, which correspond to the π→π* transition of the C=C segment [20] and the n→π* transition of the C=O bond [21]. The decoration of N-CQDs is determined by Fourier Transform Infrared (FTIR) as shown in Figure 3e, the three sharp peaks at 3465, 2920 and 2850 cm^−1^ are attributed to the –OH stretching vibration, C-H stretching vibration and C-H binding vibration. The presence of the C–N stretching vibration at 1160 cm^−1^ indicates that the N atoms have been successfully doped into the hexagonal structure of N-CQDs [15] The absorption bands located at 3415 and 615 cm^−1^ are related to the N–H stretching vibrations. Besides, there are several other characteristic peaks centered at 1617, 1380, and 1045 cm^−1^, which are ascribed to C=C stretching, C=O stretching, and C–O–C stretching, respectively. These results suggest that substantial functional groups have been formed on the surface of the N-CQDs, which is beneficial for the carrier extraction at TiO_2_/N-CQDs interfaces [20] Figure 3f shows the corresponding photoluminescence (PL) spectra of N-CQD solutions under various excitation wavelengths (λex) ranging from 310 to 550 nm. It is found that the peak intensity and position of emission wavelengths (λ_em_) are gradually changed by tuning the values of excitation light, which is mainly attributed to the surface states of the functional oxygenic groups, leading to various trapping states with energy levels within the energy gap [10,22]. Furthermore, as shown in Figure 3g, N-CQDs demonstrate an intriguing up-conversion property: when the excitation wavelengths are changed from 700 to 1000 nm, the PL emission peaks are located in the range from 450 to 540 nm, which can be arising from the an up-conversion transition or a multi-photon absorption process [23], in which the N-CQDs harvest two or more photons and subsequently drop to the valence band to emit a higher energy light. When applied in a photovoltaic device, the up-converted behavior is beneficial for maximizing the light absorbance and realizing the efficiency enhancement.

**Figure 4:**
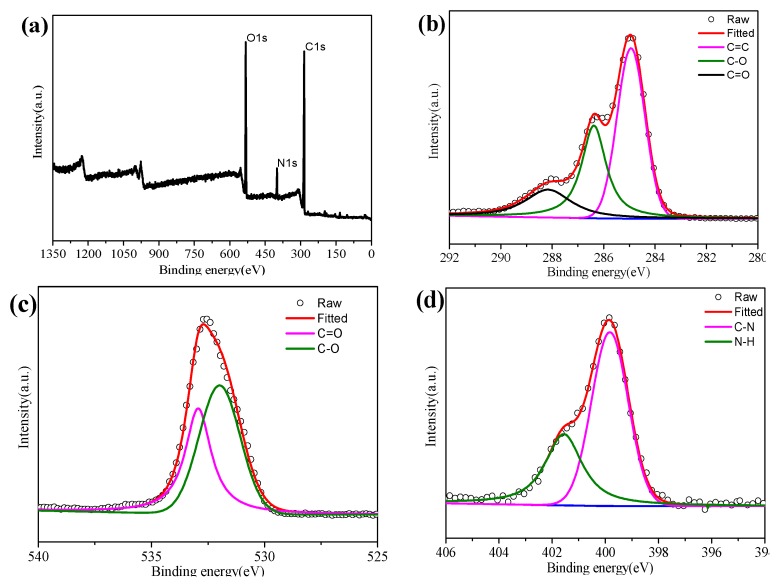
XPS survey scan of N-CQDs on the Si substrate. XPS full scan spectrum (**a**) and XPS high-resolution survey scan of (**b**) C1s, (**c**) N1s, and (**d**) O1s region. The heating time for N-CQDs is 2h.

The optimized structure properties of N-CQDs are responsible for their application in solar cells. Furthermore, X-ray photoelectron spectroscopy (XPS) analysis (Figure 4) is performed to verify the surface states of N-CQDs. Figure 4a shows three main components of the N-CQDs, including carbon (C1s, 284.9 eV), nitrogen (N1s, 398.9 eV), and oxygen (O1s, 530.9 eV), which is in accordance with the FTIR spectrum result. The C1s spectrum (Figure 4b) is fitted to three main peaks at 284.9 eV, 286.3 eV, and 288.2 eV, which are assigned to bonds of C–C, C–N/C–O, and C–O, respectively. The N1s spectrum of the N-CQDs (Figure 4c) can be split into two peaks at 399.8 and 401.6 eV, revealing the presence of a nitrogen element in the form of C–N and N–H bonds. Figure 4d denotes the O1s spectrum, fitting into two peaks centered at 531.8 and 532.9 eV for the C=O and C–OH/C–O–C groups, respectively. Until now, it can be seen that the hydrophilic functional groups have been formed on the surface of the N-CQDs during the hydrothermal process, which in turn determines their optical properties [19].

**Figure 5:**
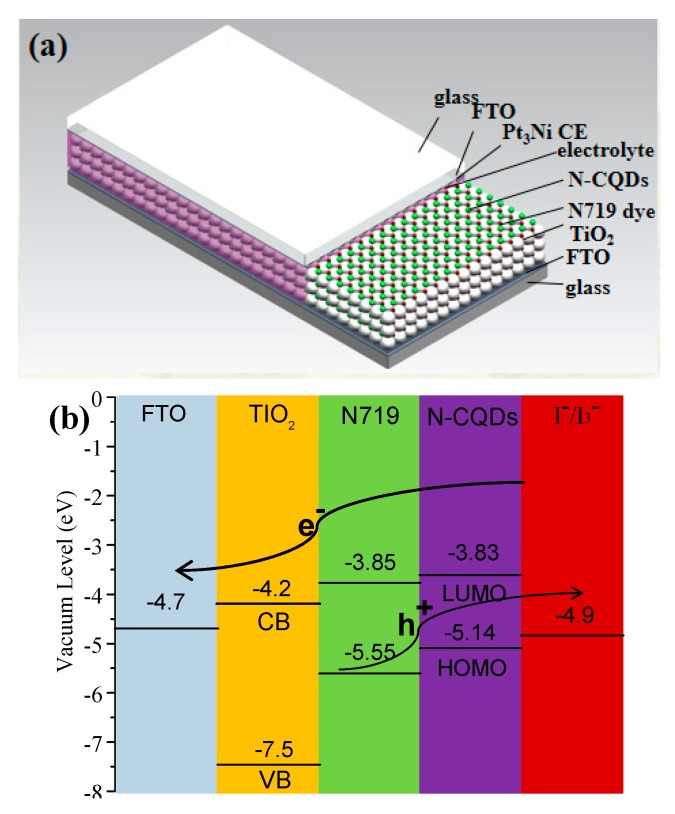
(**a**) The schematic diagram of the co-sensitized solar cells and (**b**) energy level distribution and charge transfer processes at the interface.

Due to the excellent photo excitation properties and up-conversion ability of N-CQDs, these QDs can be used in co-sensitized solar cells with dye N719. The co-sensitized solar cells were assembled by a fluorinated tin oxide (FTO) supported N719/N-CQDs co-sensitized photoanode and a Pt_3_Ni alloying counter electrode (CE) with liquid I^−^/I^3−^ couple as electrolyte, as shown in Figure 5a. To understand the effective electron transfer in this co-sensitized solar cell, the energy level distribution of TiO_2_ and N-CQDs via the cyclicvoltametry (CV) method (Figure S5) is illustrated in Figure 5b. It is obvious that the band alignment of the N-CQDs perfectly matches with the energy bands of N719 dye. Meanwhile, the Lowest Unoccupied Molecular Orbital (LUMO) energy level of N-CQD (−3.83 eV) is more positive than the conduction band (CB) energy level of TiO_2_ (−4.2 eV), and the HOMO energy level of N-CQD (−5.14 eV) is more negative to the redox potential of I^−^/I^3−^ electrolyte (−4.9 eV), The energy level of N-CQDs with different heating times is given in Figure S6, relatively, the N-CQDs at heating 2 h are more compatible with the connecting layer, confirming the effective separation of the electron hole couple.

**Figure 6:**
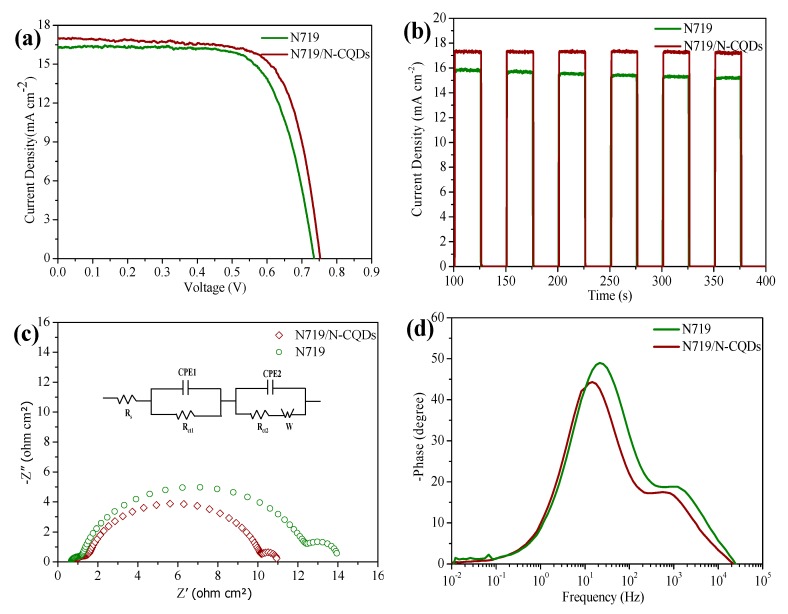
(**a**) Characteristic *J*–*V* curves for the solar cells; (**b**) The on-off switches for an optimized solar cells with or without N-CQDs. (**c**) Nyquistand. (**d**) Bode EIS phase plots of the solar cells. The insert presents the equivalent circuit.

The light absorption range of the absorber to solar spectrum performs an indispensable role for its application in advanced photovoltaics. Figure S7 plots the UV-Vis absorption spectra of TiO_2_, N719/TiO_2_ and N-CQDs/N719/TiO_2_ photoanodes. Obviously, the absorption ability of the N-CQDs/N719/TiO_2_ electrode can be significantly enhanced owing to the response of N-CQDs to the visible light as well as near-infrared light via their up-conversion ability [24]. The (*J*–*V*) characteristics curves of the co-sensitized solar cells are shown in Figure 6a, and their photovoltaic parameters are summarized in Table 2. After adding N-CQDs, a significant improvement in photovoltaic performance can be observed for the case of N-CQD modification. There is an increment of 19 mV in the Voc. Meanwhile, the *J*_sc_ and FF are both increased to 17 mA cm^−2^ and 72% from 16.5 mA cm^−2^ and 70% for the TiO_2_-N719 dye-based anodes. Finally, a PCE of 9.15% has been achieved, which is much higher than the 8.5% efficiency of the device without N-CQDs. The error bars of the DSSCs with or without N-CQDs are supported in Figure S8, showing the standard deviation among 15 measurements for the same device. The mechanism behind this phenomenon can be attributed to the following reasons: (1) The energy levels of N-CQD match well with the energy levels of TiO_2_ and the reduction potential of I^−^/I^3−^, therefore, the electrons can inject into the TiO_2_ but not into the electrolyte, serving the function of an electron-blocking layer that is typically adopted in an inorganic solar cell [25]. (2) There is a FRET (Förster resonance energy transfer) mechanism (see Figure S9). Under photoexcitation, the N-CQDs can serve as charge transfer antennas to receive and transmit energy to N719 dye. This coupling shows the synergistic interaction and significant enhancement of the light-absorption ability of the device [26]. (3) The optimized CQDs exhibit the excellent up-convention luminescent property, broadening the absorption spectrum of the device and enhancing the light-harvesting ability in the long-wavelength range [27]. For a comparison, Table S3 provides a detailed comparsion of the data that have been reported previously about the co-sensitized solar cells. These devices fabricated with the CQDs without doping shows only an efficiency of <9%.

Fast response is a key factor for practical applications of co-sensitized solar cells in electrical equipment. As shown in Figure 6b, the photocurrent signal follows the illumination light abruptly. Interestingly, the photocurrent amplitude of the device with N-CQDs is more steady than the device without N-CQDs after multiple cycles. A mechanism behind this phenomenon is that the N-CQDs can generate more electrons and then inject them into the TiO_2_, which could better maintain the *J*_sc_ in the solar cell [26]. Figure 6c shows the Nyquist EIS plots for the co-sensitized and dye-only solar cells and an equivalent circuit for fitting EIS plots. The corresponding electrochemical parameters are summarized in Table S4. We can find that the attached N-CQDs can decrease the TiO_2_/dye/electrolyte interface resistance (Rct), the co-sensitized solar cells has lower Rct value of 8.13 ohm compared to that for pristine DSSC (11.55 ohm), indicating that the charge recombination is effectively restricted, resulting in a higher *V*_oc_ value in the co-sensitized solar cells. The Bode spectrum (Figure 6d) can also offer consistent results. The calculated electron lifetime (*τ* = 1/2π*f*_max_, *f*_max_ is peak frequency) on the N719/N-CQDs electrode is much longer than that of the N719 electrode (11.4 ms versus. 7.2 ms). The longer electron lifetime is due to the suppression of charge recombination by N-CQDs, which leads to a higher charge collection efficiency in the device [26].

## 3. Conclusions

In summary, we have successfully prepared the N-CQDs for the benefit of being used in photovoltaic devices via a varying heating time. The optimized of N-CQDs were characterized by comprehensive measurements, including TEM, XRD, Raman, UV-Vis, FTIR, PL, and XPS. Meanwhile, we also fabricated a co-sensitized solar cell with N719 dye and optimized N-CQDs as sensitizers. The constructed co-sensitized solar cell yields a power conversion efficiency of 9.15% under AM 1.5G, which is much higher than that of 8.5% without N-CQDs. The results suggest that the N-CQDs are not only a suitable donor material, but also perform an important role in the process of electronic transport. Further work on optimizing the properties of N-CQDs and the interaction mechanism with corresponding dye molecules will provide insights pertaining to the usage of N-CQDs in new-generation solar cells.

## Figures and Tables

**Table 1:** Photovoltaic parameters of N-CQDs sensitized solar cells obtained from *J–V* measurements. *J*_sc_: short-circuit current density, *V*_oc_: open-circuit voltage, *FF*: fill factor.

Heating Time (h)	*J*_sc_ (mAcm^−2^)	*V*_oc_ (mV)	*FF*	*H* (%)
Raw carbon	0.023	270	0.51	0.003
0.5	0.122	382	0.49	0.023
1	0.185	400	0.54	0.040
2	0.369	428	0.56	0.089
4	0.295	428	0.52	0.066
8	0.246	410	0.48	0.049
16	0.179	387	0.49	0.034

**Table 2:** Photovoltaic parameters of co-sensitized solar cell.

Sample	*J*_sc_ (mA cm^−2^)	*V*_oc_ (mV)	*FF*	*H* (%)
N719	16.5	734	0.7	8.5
N719+N-CQDs	17	753	0.72	9.15

## Supplementary Materials

The following are available online at https://www.mdpi.com/2079-4991/10/3/460/s1, Figure S1: The chemical structure of the N719 dye; Figure S2: The high resolution TEM micrograph of N-CQDs at 0.5 h, 2 h and 8 h heating times; Figure S3: Average power conversion efficiencies of various N-CQDs tailored QDSSCs; Figure S4: The high resolution TEM micrograph of optimized N-CQDs; Figure S5: Cyclic voltammograms for N-CQDs at 2 h heating time; Figure S6: The energy level of N-CQDs with different heating times; Figure S7: The UV-vis diffuse reflectance absorption spectra of TiO2, TiO2/N719 and TiO2/N719/N-CQDs; Figure S8: Error bars of the DSSCs with or without N-CQDs, the standard deviation among 15 measurements for the same device; Figure S9: The absorption spectrum of N719 dye (black line) and emission spectra of N-CQDs (blue line) upon excitation at 390 nm, in order to support the FRET mechanism; Table S1: The comparsion date about as-prepared CQDs outstanding advantages other kinds of carbon dots sensitized solar cells reported previously as light absorbers; Table S2: Electrochemical parameters for N-CQDs sensitized solar cells; Table S3: The comparsion date about the co-sensitized solar cells reported previously; Table S4: Electrochemical parameters for co-sensitized solar cells.
