# Supplementary material for: Precise Surface State Control of Carbon Quantum Dots to Enhance Charge Extraction for Solar Cells
Source: Nanomaterials (Basel). 2020 Mar 4;10(3):460. doi: 10.3390/nano10030460 (PMC7153469; doi:10.3390/nano10030460)
Supplement: Supplementary file 1 [file nanomaterials-10-00460-s001.pdf]

Supporting documents

1. Experimental section

1.1 Preparation of fluorescent N-CQDs

N-CQDs were synthesized using natural chitosan powders as the precursor via a traditional hydrothermal method. In a representative synthesis, 0.5 g of chitosan powders was dissolved in 70 mL of pure water. Then the resultant solution was transferred into a 100 mL Teflon-lined autoclave. The reaction temperature was controlled at 180 ^{o}C for 0.5, 1, 2, 4, 8 and 16 h to obtain N-CQD aqueous solutions. The N-CQDs powders was obtained by freeze-drying for 50 h, and the obtained N-CQDs powders were re-dispersed in ethylalcohol at a concentration of 50 mg mL^{-1} for use.

1.2 Preparation of co-sensitized TiO_2 photoanodes

The TiO_2 films were prepared according to the previously reported method[1]. Subsequently, the resultant TiO_2 films were immersed for 16hrs in the N-CQDs aqueous solution at room temperature to obtained the N-CQDs sensitized TiO_2 photoanodes. For the N-CQDs/N719 co-sensitized photoanodes, the resultant TiO_2 films were first immersed for 24h in a 0.5 mM N719 ethanol solution, and the sensitized electrodes were washed with ethanol and dried under a N_2 stream. The 0.1mL as-prepared N-CQDs (50 mg mL^{-1}) were deposited onto the top of the N719

sensitized electrode via a spin-coating method with the speed of 300 rpm[2].

1.3 Fabrication of N-CQDs sensitized solar cells and co-sensitized solar cells

N-CQDs sensitized solar cells are composed of a mesoporous TiO_2 film, which is sensitized with the optimized N-CQDs, a I^-/I_3^- redox electrolyte and a Pt_3Ni alloy counter electrode (CE), the preparation of Pt_3Ni alloy CE and liquid electrolyte were based on previously report[3]. A liquid electrolyte composed of 0.1 M tetraethylammonium iodide, 0.1 M tetrabutylammonium iodide, 0.1 M LiI, 0.1 M NaI, 0.1 M KI, 0.05 M I_2 and 0.05 M 4-tertbutyl-pyridine in acetonitrile. Each co-sensitized solar cell device was built by sandwiching a I^-/I_3^- redox electrolyte between a N-CQD/N719 co-sensitized TiO_2 photoanode and a Pt_3Ni alloy CE.

1.4 Characterization

The morphology of the N-CQDs was investigated by transmission electron microscopy (TEM, FEI Tecnai G-20). Fourier transform infrared (FT-IR) spectra of optimized N-CQDs were measured to investigate specific molecule-groups information (Bruker VERTEX70). The crystal structure of N-CQDs was analyzed by a X-ray diffraction (SHIMADZU XRD-6000, corporation, Japan) with Cu radiation (λ = 0.154 nm) at 40 kV and 40 mA. Raman spectrum (Perkin Elmer, UK) were employed to characterize the N-CQDs structures at room temperature. The ultraviolet-visible absorption characterization was carried out on a UV-8000PC UV-Visble

spectrophotometer. The fluorescence spectra were measured with a fluorescence spectrometer (F-7000, Hitachi High Tech CO, Japan) with various excitation wavelengths. The X-ray photoelectron spectroscopy (XPS, Escalab 250Xi system) were conducted to analyze the surface properties. The energy levels of N-CQDs were performed via a cyclic voltammetry (CV) method using a standard three-electrode system, with a N-CQDs/TiO_2 photoelectrode, a saturated Ag/AgCl reference electrode and a Pt mesh counter electrode. The CV curves were scanned from -1.2 to 1.2 V at a scanning rate of 50 mV s^{-1}. The LUMO and HOMO levels were calculated according to the following formula: E_{LUMO} = -e (E_{red} + 4.4) V, E_{HOMO} = -e (E_{ox} + 4.4) V.

The photocurrent-voltage (*J-V*) measurements of devices were performed by using a CHI660E electrochemical workstation under irradiation of a simulated AM1.5 light illumination (SS-20FL Solar simulator, 100 mW cm^{-2} calibrated by a standard silicon solar cell). The active area is 0.25 cm^2 for both solar cells. Each *J-V* curve was repeated measured at least 15 times to eliminate experimental errors. The Electrochemical impedance spectroscopy (EIS) was measured by using the same equipment and setup,

2. Supporting Figures and Tables

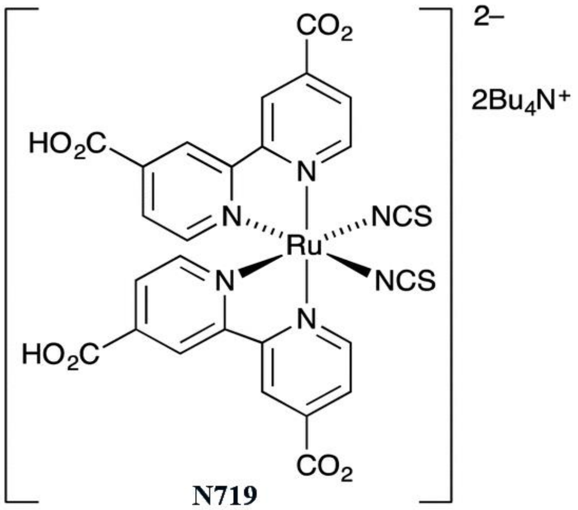

Fig. S1 the chemical structure of the N719 dye

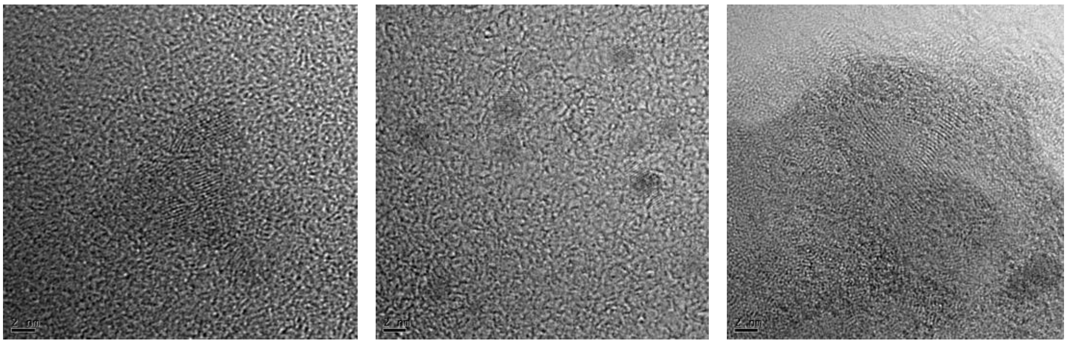

0.5h   2h   8h

Fig. S2 The high resolution TEM micrograph of N-CQDs at 0.5h, 2h and 8h heating times.

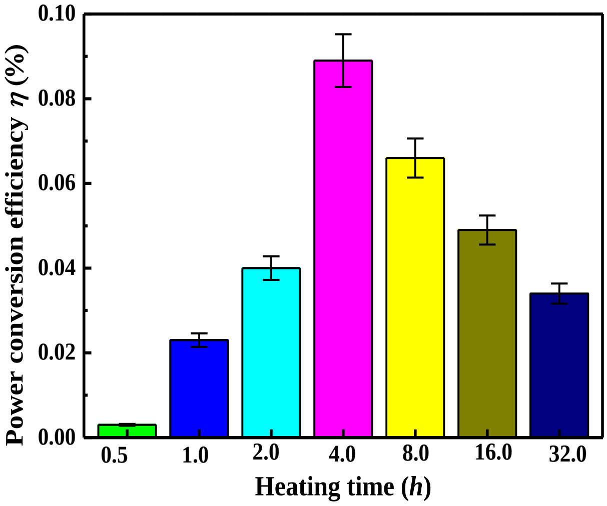

Fig. S3 Average power conversion efficiencies of various N-CQDs tailored QDSSCs. Error bars show the standard deviation among 15 measurements for the same device.

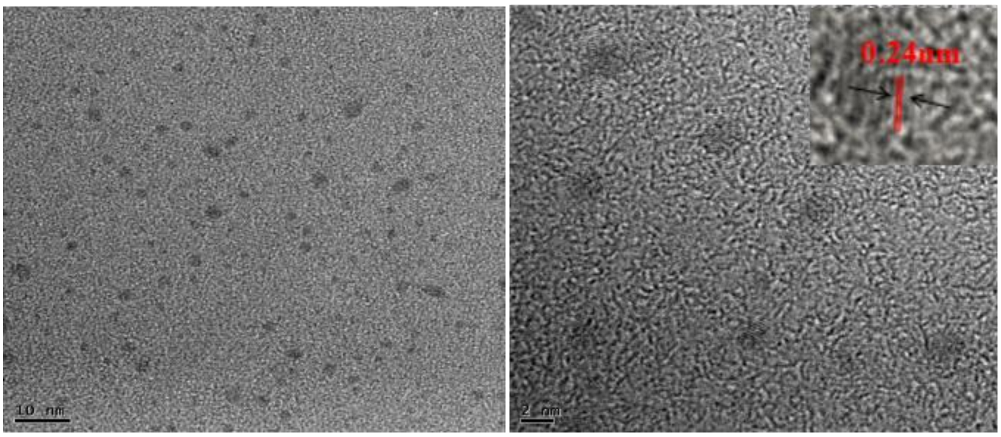

Fig. S4 The high resolution TEM micrograph of optimized N-CQDs.

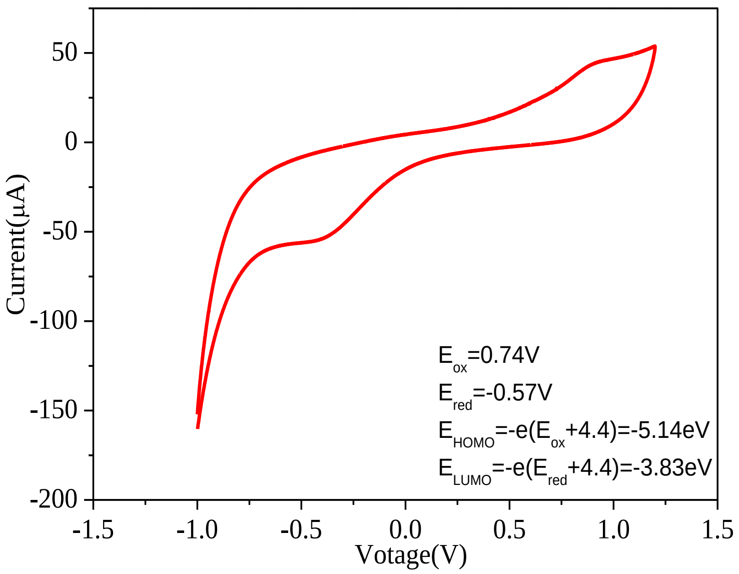

Fig. S5 Cyclic voltammograms for N-CQDs at 2h heating time.

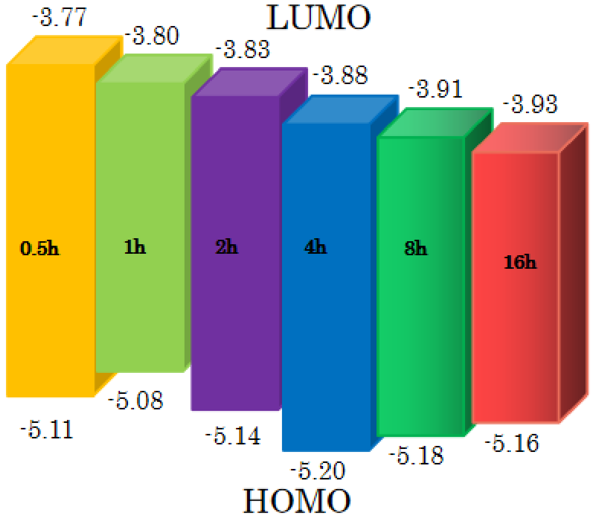

Fig. S6 The energy level of N-CQDs with different heating times.

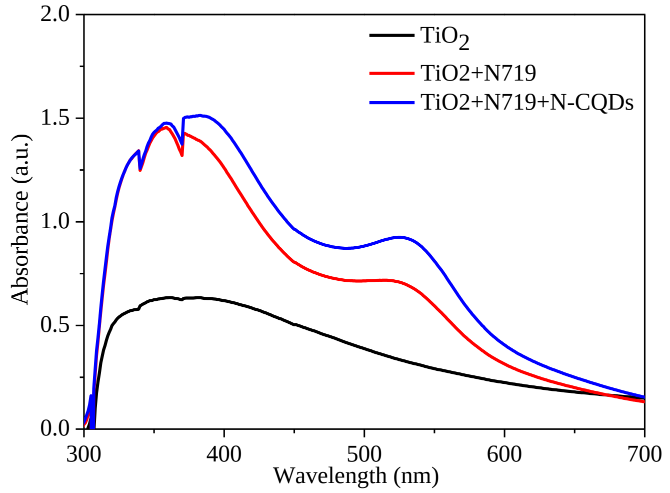

Fig. S7 The UV-vis diffuse reflectance absorption spectra of TiO_2, TiO_2/N719 and TiO_2/N719/N-CQDs.

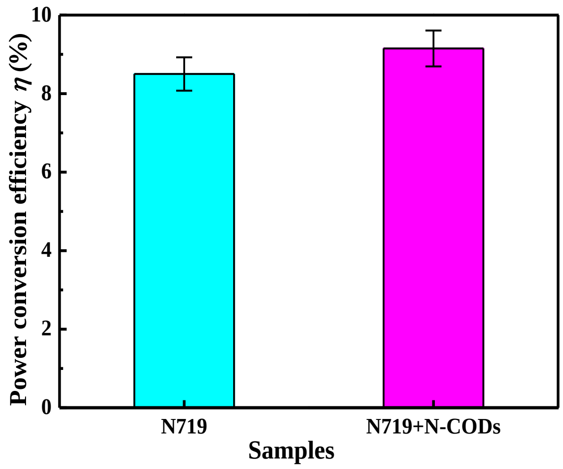

Fig. S8 Error bars of the DSSCs with or without N-CQDs, the standard deviation among 15 measurements for the same device.

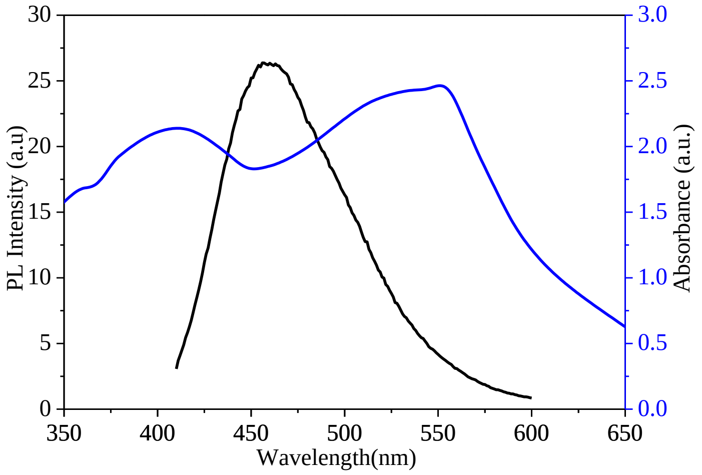

Fig. S9 The absorption spectrum of N719 dye (black line) and emission spectra of N-CQDs (blue line) upon excitation at 390 nm, in order to support the FRET mechanism.

Table S1 The comparsion date about as-prepared CQDs outstanding advantages other kinds of carbon dots sensitized solar cells reported previously as light absorbers

Teams	Carbon resource	Preparation methods	V_{oc} (mV)	J_{sc} (mA)	*FF* (%)	η (%)
Yan *et al*[4]	Graphenes	solubilization strategy	480	0.2	0.58	0.056
Mirtchev *et al*[5]	γ-butyrolactone	Carbonization with strong acid	370	0.516	0.59	0.13
Xiaochen Guo *et al*[6]	Bee Pollens	Hydrothermal	461	0.33	0.73	0.11
Our group	chitosan	Hydrothermal	428	0.369	0.56	0.089

Table S2 Electrochemical parameters for N-CQDs sensitized solar cells. R_s is series resistance; R_{ct1} refers to charge-transfer resistance at Pt counter electrode/electrolyte interface; R_{ct2} corresponds to charge-transfer resistance at photoanode/electrolyte interface. The tail in the low frequency region indicates Warburg diffusion process of redox couples (W).

Heating time (h)	R_s (ohm cm^2)	R_{ct1} (ohm cm^2)	R_{ct2} (ohm cm^2)	W (ohm cm^2)
Raw carbon	12.6	46.8	1240	145
0.5h	5.71	23.1	457	63.2
2h	2.52	16.7	200	24.5
16h	2.50	42.7	358	43.8

Table S3 The comparsion date about the co-sensitized solar cells reported previously

Teams	Absober	V_{oc} (mV)	J_{sc} (mA)	FF (%)	η (%)
Zhu *et al*[7]	Dye+PEG-CQDs	708	17.01	0.68	8.19
Fang *et al*[8]	Dye+PEG-GQDs	660	14.07	0.59	6.10
Mihalache *et al*[9]	N3+GQDs	583	5.58	0.66	2.15
Our group	Dye+N-CQDs	753	17	0.72	9.15

Table S4 Electrochemical parameters for co-sensitized solar cells. R_s is series resistance; R_{ct1} refers to charge-transfer resistance at Pt counter electrode/electrolyte interface; R_{ct2} corresponds to charge-transfer resistance at photoanode/electrolyte interface. The tail in the low frequency region indicates Warburg diffusion process of redox couples (W).

Sample	R_s (ohm cm^2)	R_{ct1} (ohm cm^2)	R_{ct2} (ohm cm^2)	W (ohm cm^2)
N719	0.87	0.67	11.13	1.62
N719+CQDs	0.65	0.55	8.55	0.82

References

[1]Tang, Q.W.; Zhang, H.H.; Meng, Y.Y.; He, B.L.; Yu, L.M. Dissolution Engineering of Platinum Alloy Counter Electrodes in Dye-Sensitized Solar Cells. Angew. Chem. Int. Ed. 2015, 54, 11610–11614.

[2] Kundua, S.; Sarojinijeeva, P.; Karthick, R.; Anantharaj, G.; Saritha, G.; Bera, R.; Anandan, S.; Patra, A.; Ragupathy, P.; Selvaraj, M.; et al. Enhancing the Efficiency of DSSCs by the Modification of TiO2 Photoanodes using N, F and S, co-doped Graphene Quantum Dots. Electrochim. Acta 2017, 242, 337–343.

[3] Tang, Q.W.; Duan, Y.Y.; He, B.L.; Chen, H.Y. Platinum Alloy Tailored All-Weather Solar Cells for Energy Harvesting from Sun and Rain. Angew. Chem. Int. Ed. 2016, 128, 14624–14628.

[4] Yan, X.; Cui, X.; Li, B.; Li, L,S. Solution-Processable Graphene Quantum Dots as Light Absorbers for Photovoltaics. Nano Lett. 2010, 10, 1869-1873.

[5] Mirtchev, P.; Henderson, E,J.; Soheilnia, N.; Yip, C,M.; Ozin, G,A.; Solution phase synthesis of carbon quantum dots as sensitizers for nanocrystalline TiO_2 solar cells. J. Mater. Chem. 2012, 22, 1265-1269.

[6] Guo, X.; Zhang, H.; Sun, H.; Tade, M,O.; Wang, S.; Green Synthesis of Carbon Quantum Dots for Sensitized Solar Cells. ChemPhotoChem 2017, 1, 1-5.

[7] Zhu, W,L.; Duan, J,L.; Duan, Y,Y.; Zhao, Y,Y.; Tang, Q,W. Efficiency enhancement of hybridized solar cells through co-sensitization and fast charge extraction by up-converted polyethylene glycol modified carbon quantum dots. J. Power Sources 2017, 367, 158-166.

[8] Fang, X.; Li, M.; Guo, K. *et al*. Graphene quantum dots optimization of dye-sensitized solar cells, Electrochim. Acta 2014, 137, 634-638.

[9] Mihalachem, I.; Radoi, A.; Mihail, M.; Munteanu, C.; Marin, A.; Danila, M.; Kusko, M.; Kusko, C. Charge and energy transfer interplay in hybrid sensitized solar cells mediated by graphene quantum dots. Electrochim. Acta 2015, 153, 306-315.
